# Newborn Screening for 5q Spinal Muscular Atrophy: Comparisons between Real-Time PCR Methodologies and Cost Estimations for Future Implementation Programs

**DOI:** 10.3390/ijns7030053

**Published:** 2021-08-11

**Authors:** Vanessa Luiza Romanelli Tavares, Frederico Monfardini, Naila Cristina Vilaça Lourenço, Katia Maria da Rocha, Karina Weinmann, Rita Pavanello, Mayana Zatz

**Affiliations:** Human Genome and Stem Cell Research Center, Department of Genetics and Evolutionary Biology, Institute of Bioscience, University of São Paulo, São Paulo 05508-090, Brazil; lrtvanessa@gmail.com (V.L.R.T.); fred.monf@gmail.com (F.M.); lourenconcv@gmail.com (N.C.V.L.); krocha@ib.usp.br (K.M.d.R.); clinicamedica@ib.usp.br (K.W.); rpavanello@ib.usp.br (R.P.)

**Keywords:** spinal muscular atrophy, screening test, neonatal screening, *SMN1*, real-time PCR, genetic diagnosis

## Abstract

Since the approval of modifying therapies for Spinal Muscular Atrophy (SMA), several protocols aiming to screen *SMN1* homozygous deletion in a neonatal context have been published. However, no work has compared different methodologies along with detailed implementation costs for centers where the neonatal screening of SMA has not yet been implemented. Therefore, our work compared different qualitative real-time PCR approaches for SMA screening and the estimated costs of test implementation. Using Brazilian blood samples, the presence and absence (P/A) and melt curve protocols were analyzed. MLPA was used as a confirmatory test. The costs were calculated for the simplex and multiplex tests plus equipment. The test workflow was based on the present experience and literature report. The accuracy of the P/A protocol was 1 (95% CI 0.8677−1) using dried blood spots (DBS). The melt curve protocol also achieved 100% concordance. The consumable costs ranged from USD 1.68 to 4.42 and from USD 2.04 to 12.76 per reaction, for the simplex and multiplex tests, respectively. The equipment acquisition costs ranged from USD 44,817.07 to 467,253.10, with several factors influencing this value presented. Our work presents a framework for decision-making, with a project demonstration of the different assays that will be useful in dealing with the issues of cost and availability of reagents. Moreover, we present a literature review and discussion of important concerns regarding treatment policies. We take the first step towards a future SMA NBS pilot program where it is not yet a reality.

## 1. Introduction

5q Spinal Muscular Atrophy (SMA, MIM 253300, 253550, 253400, and 271150) is an autosomal recessive neuromuscular disorder characterized by the degeneration of α-motor neurons resulting in muscle weakness and atrophy. Its incidence is estimated in 1:6000–11,800 live births. The prevalence is 1 to 2/100,000 due to the high death rate in the most frequent type of SMA (type 1), which accounts for about 60% of SMA cases [1,2,3,4]. The disease presents a wide range of clinical expressivity, being traditionally classified according to the age of onset and achieved motor function (types 0–4) when considering the natural history of SMA [5,6]. Some classify the disease into non-sitters, sitters, and ambulatory [7].

SMA is caused by the homozygous loss of function of the survival motor neuron 1 gene telomeric (*SMN1*, MIM: 600354). About 95% of patients have homozygous deletions or gene conversions of *SMN1* into *SMN2* [8]. Compound heterozygous mutations are found in 3–5% of patients, where one allele of *SMN1* has a deletion and the other presents a subtle mutation [9]. A paralog and almost identical gene (with just five nucleotides of difference from *SMN1*), named *SMN2*, has been described as the major disease-modifying gene in SMA (reviewed in reference [10]).

During the last decades, several therapeutic approaches have been developed aiming to modify the natural course of this severe disease [11]. With therapies already approved [11,12,13,14], research efforts for an earlier diagnosis and pre-symptomatic treatment have rapidly increased. Patients with SMA type 1 may lose over 95% of their motor functions in the first six months of age [15], and because of this rapid denervation and progressive weakening [16], a therapeutic intervention immediately after diagnosis and before the onset of symptoms is expected to produce better outcomes. For instance, in the USA, SMA was included in the recommended uniform screening panel (RUSP), and several states have already implemented the program. Similarly, Australia, Germany, Belgium, Taiwan, China, and Japan have developed pilot SMA newborn screenings (NBS) and follow-up studies [17,18,19,20,21,22,23].

The majority of screening tests are based on real-time PCR. The protocol modifications include the targeted pre-amplification of the *SMN* gene before the real-time PCR and the use of *SMN2* blocker primers, locked nucleic acid (LNA) *SMN1* primers/probes, and minor groove binder (MGB) probes [20,24,25,26,27,28]. Moreover, others have developed different strategies, as is the case of Ontario Province with the multiplex MassARRAY of SMA along with the genes for hearing loss and severe combined immunodeficiency [29].

The major challenges of SMA screening tests are related to the DNA quality and quantity and cross-reaction of *SMN1* with *SMN2*. This seems to be successfully circumvented through different DNA extraction methods and LNA primers/probes and/or well-established cutoffs [22]. The NBS methodologies implemented allow for the detection of homozygous deletions in *SMN1* corresponding to about 95% of SMA patients, but about 3–5% of patients may be missed due to the presence of subtle mutations along the *SMN1* gene [26,30,31].

So far, studies aiming at SMA NBS within a Latin American setting have been lacking. Our work presents a framework for decision-making, with a project demonstration of different assays that will be useful for dealing with the issues of cost and availability of reagents. Moreover, we included a literature review to discuss the workflow needed for a SMA NBS program up to the clinician’s availability and newborn’s treatment that should be a point of careful revision in countries just starting. With this strategy, we take the first step in the evaluation of possible assays toward a pilot program to be run in Brazil and outline some of the next steps and considerations in its design.

## 2. Materials and Methods

### 2.1. Study Population and DNA Samples

In the present study, 129 blood samples were included. Of these, 54 samples had their *SMN1*/*SMN2* genotype previously obtained by Multiplex ligation-dependent probe amplification (MLPA) and recorded at our center (hereafter referred to as retrospective cohort). The other 75 samples were recruited through convenience sampling (hereafter referred to as prospective cohort) and included blood samples collected from SMA and non-SMA patients (20 with a clinical hypothesis of SMA, 40 SMA relatives and possible carriers, and 15 non related and non-SMA individuals). The DNA samples were extracted from peripheral blood collected in a 4-mL tube containing EDTA through Autopure LS (Qiagen/Gentra, Hilden, Germany) (retrospective cohort) or Biorobot QIAsymphony (Qiagen, Hilden, Germany) (prospective cohort), according to the manufacturer’s protocols, and stored at −20 °C. Additionally, individuals either from the retrospective or prospective cohort (*n* = 26) were randomly assigned to also have their blood collected on S&S 903 filter paper (kindly supplied by Instituto Jô Clemente, São Paulo, Brazil) for the sample source validation. These samples will be hereafter referred to as the mixed cohort (Supplementary Table S1). Dried blood spots (DBS) were stored in low-permeability zip-lock bags with silica gel desiccant packs at 4 °C (up to six months) and at −20 °C if longer prior to DNA extraction. The DNA was obtained from DBS, as described in Strunk et al. [32]. Briefly, a single 3.2-mm punch was incubated at room temperature for 15 min in 100 uL of 10 mM of NaOH. After, the liquid was washed out, and the procedure was repeated; the punch was incubated at 99 °C for 15 min in 50 uL of 10-mM NaOH. All extracted DNA samples could be stored at −20 °C, but the use of freshly non-diluted DNA was recommended. Three 3.2-mm discs were punched from a blank paper filter between each sample to avoid cross-contamination. 

The study was approved by the Ethics Committee in Research (ECR)—Human Beings of the Institute of Bioscience at USP, São Paulo, Brazil (CAAE: 86638318.2.0000.5464). All the patients, parents, and guardians provided signed informed consent accordingly. 

### 2.2. Real-Time PCR 

#### 2.2.1. Presence and Absence (P/A) Assay—In-House *SMN1* Standardized Test

A TaqMan real-time PCR assay was conducted as a P/A analysis to detect only the homozygous deletion of *SMN1* exon 7 (SMA patients) based on the failure of *SMN1* exon 7 amplification. The primers and probe sequences of *SMN1* and RNase P gene (internal control) were essentially as previously described, as well as the reaction conditions [33] but using 2 uL of freshly non-diluted DNA (when the sample source was peripheral blood collected in an EDTA tube) or 6 uL (when using DNA extracted from DBS, with the volume determined during the in-house validation—data not shown). No DNA measurement/quantification was necessary. All the samples were analyzed in duplicates, and each plate contained a negative (non-SMA, ≥1 *SMN1* copy) and a positive (SMA, 0 copies of *SMN1*) control sample, besides a blank reaction and paper blank controls when appropriate. The thermocycling parameters were optimized in QuantStudio 5 (Thermo Fisher Scientific, Waltham, MA, USA) equipment as follows: a pre-read stage at 50 °C for 2 min and hold stage at 95 °C for 10 min, followed by 40 cycles of 95 °C for 15 s and 64 °C for 1 min; then, a post-read stage at 60 °C for 30 s. Fluorescence was acquired in the 50 °C, 64 °C, and 60 °C steps (data automatically analyzed from the pre-PCR and post-PCR reads). The data were extracted using QuantStudio Design & Analysis software v1.4.2. The Cq thresholds were automatically set. The Cq cutoff values for RNase P and *SMN1* were established determining the 2.5–97.5 percentile. The 97.5 upper limit was used to set the cutoffs. Samples above the cutoff and/or with discordant replicates were assigned as inconclusive and retested with a fresh DNA extraction. 

#### 2.2.2. Melt Curve Assay—Commercial Simplex *SMN1* Test

The Salsa MC002 SMA Newborn Screening assay, kindly supplied by MRC-Holland (Amsterdam, The Netherlands) through Citogem (São Paulo, Brazil), was evaluated using 26 DBS cards. The assays were conducted as indicated by the manufacturer, including the DNA extract procedure. The reactions were run in duplicates in QuantStudio 5 (Thermo Fisher Scientific, Waltham, MA, USA). The analyses were conducted visually in QuantStudio Design & Analysis software v1.4.2., as indicated by the manufacturer.

### 2.3. Multiplex Ligation-Dependent Probe Amplification (MLPA)—Standard Test

The copy numbers of *SMN1* and *SMN2* were determined using SALSA MLPA P021 SMA probemix and a SALSA MLPA EK1 reagent kit (MRC-Holland, Amsterdam, The Netherlands). The protocol was performed according to the manufacturer’s instructions. The amplification products were quantified on an ABI 3730 DNA analyzer (Applied Biosystems-Thermo Fisher Scientific, Waltham, MA, USA), and the data was analyzed using GeneMarker v1.95 (SoftGenetics, State College, PA, USA).

### 2.4. Statistical Analysis

The analysis was conducted with R programming language [34]. The data distribution was evaluated with Q–Q plots and the Shapiro–Wilk test. The descriptive statistics were reported accordingly (means and standard deviations or medians and interquartile ranges (IQR)). A Kruskal–Wallis with Dunn’s post-hoc test was performed to compare the non-normal data. A *p*-value < 0.05 was considered to be significant. The sensitivity, specificity, and accuracy were obtained with a 95% confidence interval (CI).

## 3. Results

### 3.1. Presence and Absence (P/A) Protocol

**Figure 1:**
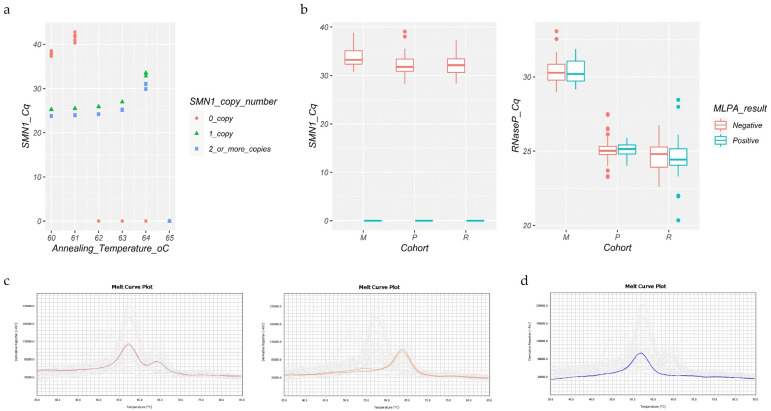
(**a**) Temperature gradient. The specificity for *SMN1* over *SMN2* is observed at 62 °C, 63 °C, and 64 °C. The highest specificity was chosen for the assay. The *SMN1* copy number refers to the MLPA genotypes. (**b**) Box plots of the Cq values obtained in the real-time PCR test. The box colors indicate the result obtained through MLPA. The data include inconclusive samples. Significant differences were observed for the medians of the *SMN1* Cq values between the mixed compared to the retrospective and prospective cohorts (*p*-values < 0.0005). The RNase P Cq medians were different among all the comparisons (*p*-values < 0.0005). M, mixed cohort; P, prospective cohort; R, retrospective cohort. (**c**,**d**) The melt curve assay. Representative plot of two negative samples showing *SMN1* peaks in both images and a *SMN2* peak in the right one (**c**); representative plot of a positive sample result showing just the *SMN2* peak (**d**).

The analytical specificity of *SMN1* was obtained at the annealing temperature of 64 °C (Figure 1a). The P/A protocol (standardized in-house) was firstly evaluated in 54 retrospective samples genotyped via MLPA (retrospective cohort). No samples were inconclusive. Overall, the test achieved 100% concordance with the *SMN1* genotypes ascertained via MLPA (27 true-negative and 27 true-positive samples). The RNase P Cq median was 24.50 (IQR 1.27) for the negative and positive samples, while the *SMN1* Cq median was 32.13 (IQR 2.82) for the negative samples. In this cohort, the upper limit was set at Cq 26.70 for RNase P and at Cq 36.72 for *SMN1*. 

Using the established annealing temperature, we performed a blind test on 75 prospective samples (prospective cohort). The upper limits were set at Cq 26.23 for RNase P and at Cq 35.13 for *SMN1*. Two samples (2.7%) returned inconclusive and were retested using fresh DNA. The RNase P Cq median was 25.04 (IQR 0.57) for the negative and positive samples, and the *SMN1* Cq median was equal to 31.78 (IQR 2.53) for the negative samples. Overall, 18 samples were classified as true positives, 56 as true negatives, and 1 sample as a false positive, while no false negatives were observed. This resulted in a 0.9867 accuracy (95% CI 0.9279–0.9997), 1.00 sensitivity (95% CI 0.81–1), 0.9825 specificity (95% CI 0.91–1), and 0.0175 false-positive rate. The false-positive result was attributed to the low DNA quantity, placing the *SMN1* Cq values above the upper limit without interfering with the classification of the internal control.

Next, we evaluated the performance of the real-time PCR assay using 26 DNA samples extracted from S&S 903 filter paper cards (mixed cohort). The upper limits were set at Cq 32.36 for RNase P and at Cq 37.60 for *SMN1*. The blank filter papers showed Cq > 42 for *SMN1* in three wells above the established upper limit, excluding the risk of false-negative results due to cross-contamination. Two samples returned inconclusive (0.52%) and were retested. Overall, the test identified 20 true negatives and 6 true positives, without any false positives/negatives. The *SMN1* Cq median was 33.25 (IQR 2.80) for the negative samples, and the RNase P Cq median was equal to 30.27 (IQR 1.12) for the negative and positive samples. The test accuracy, sensitivity, and specificity all achieved 1.00, with a 95% CI equal to 0.8677–1, 0.54–1, and 0.83–1, respectively.

We observed that the Cq values from the mixed cohort (DNA from filter paper cards) were consistently higher for both RNase P and *SMN1* compared to either the prospective or retrospective cohorts (DNA from peripheral blood) (Kruskal–Wallis with Dunn’s post-hoc test, a *p*-value < 0.0005 for RNase P Cq among all the cohorts and a *p*-value < 0.0005 for *SMN1* Cq (retrospective versus mixed and prospective versus mixed cohorts)). This is likely due to the DNA quality/quantity associated with the filter paper samples and the DNA extraction procedure (Figure 1b).

The clinical follow-up and *SMN2* copy number of the SMA patients are described in the Supplementary Materials Figure S1.

### 3.2. Melt Curve Protocol

Aiming to compare the P/A protocol (in-house standardized) with the melt curve protocol (commercial kit), we evaluated the Salsa MC002 SMA NBS kit using samples from the mixed cohort (*n* = 26, collected on filter papers). This kit was the only SMA simplex kit commercially available up to the time of this study. Inconclusive results were not observed, with an indication of sufficient DNA input in all reactions (Q-fragment melt peak accordingly), and no cross-contamination was identified (blank paper filters with a single Q-fragment peak observed). A 100% concordance was achieved (20 true negative, with a *SMN2*-specific melt peak at 56 °C and *SMN1*-specific melt peak at 63 °C, and 6 true-positive samples, with a *SMN2*-specific melt peak at 56 °C and the absence of a *SMN1*-specific melt peak) (Figure 1c,d). As recommended by the manufacturer, the analysis was conducted manually through plot visualization. A slight variation of the indicated melt temperature was observed (Q-fragment melt peak 50.88 ± 0.27, *SMN1* melt peak 63.99 ± 0.28, and *SMN2* melt peak 57.33 ± 0.33), however, without compromising the results. 

### 3.3. Cost Estimation and Test Workflow

Based on the methodology approaches evaluated in this study, the related consumable costs were estimated in different scenarios of the real-time PCR assay for SMA. The costs for the consumable reagents/materials (including the DNA extraction step) ranged from USD 1.68 to 4.42 per reaction, considering the single reactions in a 384-well plate and 33,521 samples per month (67% of the average number of newborns alive in the state of São Paulo from 2017 to 2019 (available online: https://datasus.saude.gov.br/sobre-o-datasus/ (accessed on 5 July 2021))). Additionally to the simplex reaction scenario, multiplexing other targets, such as T-cell Receptor Excision Circles (TREC) and Kappa-deleting Recombination Excision Circles (KREC) for severe combined immunodeficiency (SCID) and agammaglobulinemia (AGAMMA), respectively, have been already described [35] and implemented in countries such as Australia. The consumable costs associated with the multiplex real-time PCR (SCID-AGAMMA-SMA) range from USD 2.04 to 12.76, an approximate USD 0.36–8.34 difference in comparison to performing the simplex SMA test alone (Table 1). 

Thus far, the majority of newborn screening tests have been biochemical, and some laboratories may need to invest in the infrastructure and equipment for molecular tests, such as the real-time PCR. The estimated costs for the equipment acquisition can range from USD 44,817.07 to 467,253.10 (R$257,250.00 to 2,682,032.78, based on USD 1.00 = R$5.74 (14 March 2021)), depending on the previous infrastructure of the laboratory. Table 2 provides an estimate for the acquisition of real-time PCR equipment; peripheral equipment (e.g., microcentrifuges, thermocyclers, and vortex mixers); and automation robots. These values may vary according to the equipment type/brand and reagent manufacturer, workflow automation, and sample demand. Moreover, the values may be influenced by negotiations with the vendors, including loans or lease agreements. It is also important to note that both the commercial and in-house tests, as well SCID and AGAMMA first-tier tests, can be run in the same real-time PCR equipment if the dyes are compatible; the peripheral equipment would also be the same. 

For a complete diagnostic workflow, a second-tier test must be included and considered in the estimated costs of a SMA newborn diagnosis. As the number of second-tier tests per year would be directly related to the population number and SMA incidence in the target population, an estimation of the internalization costs of the test would be challenging and possibly not cost-effective if the laboratory just offers MLPA on demand for SMA NBS. We can consider that a reasonable price of the MLPA test offered by some laboratories in São Paulo (Brazil) that already have a complete facility to perform MLPA is around USD 224.74 (R$ 1,290.00).

**Figure 2:**
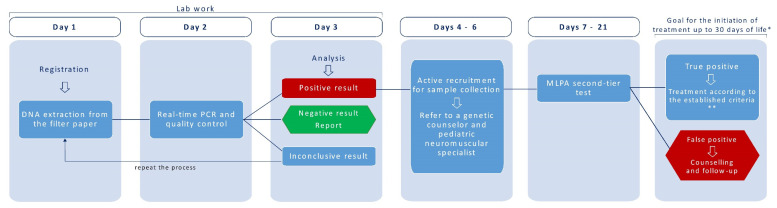
Possible workflow for SMA NBS implementation, along with patient follow-up to treatment. Days 1−3 of the laboratory work were based on a calculation of the time for the preparation of 7 to 8 plates (384-well plates) a day (about 1500 samples/day) and our current assay experience; about 2 laboratory specialists would be needed during this workflow. * Based on McMillan et al. [29]. ** International guidelines refer to treating all confirmed SMA infants with 4 *SMN2* copies or less. However, the Brazilian PCDT only includes pre-symptomatic patients with up to 3 copies of *SMN2*; this might be a point of significant revision (see Discussion for additional details). Moreover, other factors can be relevant for the clinical indication of the treatments available. The figure was based on, and modified from, McMillan et al. [29].

Regarding the time of the workflow, our study was performed in a nonautomated way, with duplicate reactions in 96-well plates. The use of single reactions can also be considered upon validation. The paper filter DNA extraction and consecutive reaction plate took about 3 h hands-on and 90 min of a plate reaction in a QuantStudio5 thermal cycler. We considered it feasible to use automated or semiautomated systems, as demonstrated by Czibere et al. [22], and the use a 384-well reaction plate, enabling the screening of up to 2000 samples per day. A possible workflow for the screening step is shown in Figure 2, along with a real example of the time to treatment [29]. 

## 4. Discussion

In the present work, we compared cost-effective *SMN1* homozygous deletion screening test through real-time PCR based either on a P/A or melt curve analysis. Using Brazilian cohorts, this represents the first SMA screening test evaluation in a Latin American setting and contributes to the understanding and mapping of possible challenges regarding the future of a national SMA NBS pilot implementation. Issues like cost estimation, clinicians’ availability, and treatment policies are important topics to be addressed in any country aiming to implement a new disease in the NBS policies.

With both the in-house P/A and the commercial melt curve test, we were able to obtain high levels of accuracy. Notably, the maximum sensitivity that such a real-time screening test can achieve is 95%, corresponding to SMA patients with *SMN1* exon 7 homozygous deletion, and a point mutation will not be identified. The only false-positive result observed was due to the poor quality of DBS, an issue already reported in other studies [33]. Most importantly, no false-negative results were observed in any approach here evaluated. Both strategies are qualitative and have internal reaction controls. In our evaluation, the main advantage of the in-house P/A approach test was the possibility of automation. Our attempt to automate the analysis of the melt curve commercial assay, although not indicated by the manufacturer, was not successful: one out of 26 samples was assigned as a false positive, because the *SMN1* melt peak was not automatically recognized by the QuantStudio software, and the negative control was not correctly assigned (data not shown). Therefore, in a high daily demand of a NBS program, an automated analysis able to provide results in a timely manner and avoid biased interpretations would be ideal. In this regard, the in-house P/A protocol could be easily exported in a file format suitable to an automated code program analysis, such as in R used in our test. Cavdarli and collaborators [36] also published an innovative automation method, using an intelligent ratio based on a reference gene and the quantification of *SMN1* exon 7, capable of detecting zero, one, or two copies of *SMN1*. However, the ethical concerns about carrier identification are still being discussed [37], and a genetic counseling follow-up would have to be applied, requiring more specialists and additional costs. On the other hand, the melt curve kit offers a set with complete positive and negative controls that, in the in-house test, have to be provided by the laboratory or acquired separately.

One of the challenges in identifying a *SMN1* homozygous deletion is to avoid a cross-reaction with *SMN2* that can lead to false-negative results. In our in-house P/A validation, this problem was avoided by setting the melting temperature at 64 °C. Our results achieved 100% concordance (retrospective and mixed cohorts). We observed a nonspecific *SMN2* late amplification at a high Cq far from the established cutoff, and therefore, this should not interfere with the ability to identify infants homozygous for *SMN1* deletion. One interesting approach for designing variant specific *SMN1* primers is to overlap by one base (variant-specific nucleotide at position 2) and to have position 3 altered, avoiding the amplification of the nontarget variant (reference [22] and personal communication). Moreover, Pan and collaborators [25] recently described LNA-modified primers that can enhance the specificity and increase the efficiency of *SMN1* amplification 6.8% compared to conventional primers. Similarly, MGB probes were also efficient in avoiding cross-hybridization of the *SMN1* probe with the *SMN2* copies [27,28].

MLPA was the second-tier test used, performed on blood samples collected in EDTA tubes, as the already established routine in our center. The use of DBS on MLPA was investigated by Strunk et al. [32] and could facilitate the workflow implementation. The authors reported 100% sensitivity and specificity of the MLPA test (P021, MRC-Holland) using crude lysates from DBS (NaOH protocol). However, this data contradicts the results achieved by Vidal-Folch and collaborators [38], where a specific copy number could not be assigned due to an ambiguous dosage quotient. They concluded that the droplet digital polymerase chain reaction (ddPCR) precision was better at low DNA concentrations obtained with DBS cards. Therefore, we recommend that MLPA using DBS should be in-house validated before the routine application.

The implementation of a NBS program can be assessed in different phases, as performed by Kellar-Guenther et al. [39]: “(1) approval to screen; (2) laboratory, follow-up and information technology capabilities; (3) education; and (4) implementation of statewide NBS”. The United States Secretary of Health and Human Services’ Advisory Committee on Heritable Disorders in Newborns and Children (ACHDNC) published a complete report regarding newborn screening for SMA. The major challenges were related to clinical care for diagnostic confirmation and insurance authorization to obtain a timely treatment and informed decisions (available online: https://www.hrsa.gov/sites/default/files/hrsa/advisory-committees/heritable-disorders/reports-recommendations/sma-nbs-implementation-report.pdf (accessed on 3 July 2021)). In Brazil, after the incorporation of nusinersen for SMA type I in Brazil’s National Healthcare System (known as the Unified Health System—in Portuguese, SUS), the efforts to provide a timely treatment for patients improved through, for example, the definition of a SMA Network of Specialized Care Centers (from Portuguese, CERAMEs) in the São Paulo state, composed by at least seven centers to be officialized (available online: https://www.al.sp.gov.br/spl/2021/06/Acessorio/1000373728_1000442241_Acessorio.pdf (accessed on 3 July 2021), and personal communication with the State Health Department/São Paulo, Brazil). Therefore, newborns diagnosed through the SMA NBS program in the future could benefit from CERAMEs for their care, follow-ups, or referral to other specialized centers. Moreover, CERAMEs would be a good example to be followed by other states in Brazil and, possibly, by the other countries of Latin America. 

Another point of concern raised by ACHDNC was related to precisely informing the *SMN2* copy number as one, two, three, four, or >four for diagnosed SMA newborns, either assessed at screening (quantitative real-time PCR) or at a confirmatory test [40,41]. The *SMN2* quantification is critical, as the *SMN2* copy number is used for treatment recommendations [40]. The majority of laboratories use MLPA, which quantifies *SMN2* copies as zero, one, two, three, or >three. MLPA is the gold standard for *SMN1* and *SMN2* copy number determination, but at least two NBS programs have also validated ddPCR to determine zero to five *SMN2* copies, offering an advantage over MLPA. (available online: https://www.hrsa.gov/sites/default/files/hrsa/advisory-committees/heritable-disorders/reports-recommendations/sma-nbs-implementation-report.pdf (accessed on 3 July 2021)).

A previous ACHDNC report mentioned the importance of funding approval for screening, something to be considered for centers around the world that are just starting the SMA NBS program. However, according to that report, 42% of the NBS program directors reported that the cost per specimen to conduct SMA screening (personnel, equipment, and reagents) was a minor barrier (summary assessed in Kemper et al. [42]). In fact, in the present estimation, the SMA simplex reaction consumable costs range from USD 1.68 to 4.42, which is similar to previous reports [17,18]. The NBS USA programs already pointed out that multiplexing SMA with other disease (such as SCID) targets is a major facilitator (50% of the answers), although SCID needs to be revalidated in the context of SMA [42]. We estimate an increase of about 0.36–8.34 dollars for multiplexing SMA with other diseases evaluated by real-time PCR (e.g., SCID and AGAMMA). The costs associated with the equipment to start the program may be mitigated via loan and leasing agreements, as well as collaborations with other NBS centers [39]. The same previous USA report estimated that about two full-time employees would be necessary to process 100,000 specimens annually for the multiplexed SCID and SMA tests and that the test can be validated within six months, which we consider to be perfectly possible. According to Kellar-Guenther et al. [39], seventeen USA states took 20 months to reach statewide implementations for SMA, and nowadays, 37 states are currently screening for SMA, representing 85% of newborn babies screened in the USA (Cure SMA, available online: https://www.curesma.org/wp-content/uploads/2021/06/NBS_Maps_Screening-and-Non-Screening_States_CKD_v6-1-2021.pdf (accessed on 3 July 2021)). Since the approval of Federal Lay 14.154 of 2021 in Brazil, Reference Services in Neonatal Screening (RSNS) will have to include the SMA in the NBS program. One of the RSNS is the Instituto Jô Clemente, São Paulo, Brazil (IJC, previously known as APAE-SP), the largest NBS laboratory in Latin America in terms of the number of exams performed. In 2019, IJC screened 395,281 children in the NBS program and performed 2,635,283 tests (IJC Activity Report 2019, available online: https://www.ijc.org.br/pt-br/sobre/transparencia-v2/relatoris/relatorios-atividades/Documents/Activities-Report-2019-web.pdf (accessed on 3 July 2021)). 

After establishing the strategy for implementation, centers have to determine the workflow and path of positive screened patients. Ontario’s pilot program has reported to have a preliminary diagnosis 7–10 days after birth (considering the samples arriving from remote sites) and a definitive diagnosis between 16 and 27 days after birth [29]. When a positive patient is identified, some NBS centers have made contact with the pediatrician listed at the time of birth to formulate a disclosure plan and, after that, brought the family to the NBS center for an evaluation, confirmatory test, and information about the SMA treatments available. In this process, some challenges have been reported, such as the availability and readiness of SMA specialists [42]. 

The time taken for diagnostic confirmation and the contact of SMA patients and physicians is a major determinant to produce better treatment outcomes. Recent studies have demonstrated the efficacy of several approaches in modifying the natural history of SMA, mainly with earlier treatments. Currently, pre-symptomatic SMA clinical trials are based on *SMN* target mechanisms, such as antisense oligonucleotide and small molecules that modify the splicing of the *SMN2* pre-mRNA, and gene replacement therapy [43]. The *SMN2* copy number provided via confirmatory MLPA or ddPCR has been used to guide patients’ treatments. Glascock and collaborators [40] updated the recommendation to immediately treat all SMA patients with four or less copies of *SMN2* based on real-world NBS data. One of the works that illustrated this decision showed a SMA patient with four copies of *SMN2* who developed SMA type 2 symptoms at the age of 8 months [41], a SMA type that may evolve into severe symptoms such as respiratory insufficiency and dysphagia [44]. The USA NBS program for SMA was reported to prevent about 50 patients undergoing ventilator dependence and about 20 deaths annually [42].

The next step in the present work should be to implement a statewide pilot program in Brazil using larger cohorts and to develop a reliable and efficient algorithm to refer patients to specialized centers capacitated in the treatment of SMA newborns before symptoms onset. The Brazilian Clinical Protocol and Therapeutic Guidelines (in Portuguese, PCDT), which regulates the antisense oligonucleotide treatment for SMA type I, already includes pre-symptomatic SMA patients but with a familial history of SMA, genetic diagnosis, and up to three copies of *SMN2* (available online: http://conitec.gov.br/images/Relatorios/2019/Relatorio_PCDT_AME.pdf (accessed on 12 March 2021)). Considering just up to three copies of *SMN2* is against the current international recommendations [40], as already discussed. The Brazilian guidelines must be revised to also make eligible patients with up to four *SMN2* copies and discuss the ethical implications of milder later-onset cases of SMA that will be identified in a NBS program [45]. Moreover, pre-symptomatic SMA patients without a previous familial history of SMA must also be eligible, considering the high carrier frequency of around one in 50, depending on ethnicity [3].

In conclusion, our study is of pivotal importance to the future of SMA NBS pilot implementations in places where they are not yet a reality, such as Latin America. Further evaluations on large cohorts will ensure the specificity of the tests performed. This work highlights the comparison of two different approaches and the attributed costs. The present cost comparison and technical scenario breakdown will contribute to the decision-making process by NBS centers regarding using simplex or multiplex tests, in-house standardized tests or those acquired commercially, and budget estimates.

## Figures and Tables

**Table 1:** Estimated costs related to the consumable reagents and materials for the SMA newborn screening first-tier test (all based on real-time PCR), including the consumables for the DNA extraction step that may differ in the commercial and in-house procedures. These costs do not take into account the second-tier assay. The calculations considered single reactions per sample in a 384-well plate and for 33,521 samples per month (see text for details). Note that some laboratories may choose to perform the test in replicates, according to the results obtained in their validation step.

	Real-Time PCR Assay Cost Per Reaction			
	Simplex Test		Multiplex Test	
	In-House Standardized	Commercial Tests	In-House Standardized	Commercial Tests
**Consumables**	USD 1.68	USD 4.42	USD 2.04	USD 5.99–12.76
	(R$ 9.64)	(R$ 25.35)	(R$ 11.68)	(R$ 34.40–73.23)

USD 1.00 = R$5.74 (14 March 2021).

**Table 2:** Estimated costs for the equipment acquisition depending on the previous infrastructure of the laboratory.

	Laboratory Status	
	Runs Genetic/Molecular Tests Other than Real-Time PCR	Does Not Run Any Genetic/ Molecular Test
**Without automation investment**	USD 44,817.07	USD 75,503.13
	(R$ 257,250.00) *	(R$ 433,387.98)
**Automation** **investment** **^†^**	USD 391,749.97	
	(R$ 2,248,644.80)	
**Total estimative**	USD 436,567.04	USD 467,253.10
	(R$ 2,505,894.80)	(R$ 2,682,032.78)

* Based on the minimal requirements of 384-well plate real-time equipment. Other versions are available and may considerably elevate the prices. ^†^ Some of the automation equipment can be used in other applications, depending on the sample demand. The laboratory must check if its current facility already attends to the needs of the real-time PCR test. USD 1.00 = R$5.74 (12 March 2021).

## Data Availability

All the data needed to evaluate the conclusions of the study are present in the paper and/or the Supplementary Materials.

## Supplementary Materials

The following are available online at https://www.mdpi.com/article/10.3390/ijns7030053/s1: Methods: Table S1. Study cohorts specified by inclusion criteria, total number, sample source, method of DNA extraction, and real time PCR assay performed. Results: Clinical follow-up and *SMN2* copy number. Figure S1: Distribution of the SMA types according to the number of *SMN2* copies.

## References

[1] Arkblad E., Tulinius M., Kroksmark A.K., Henricsson M., Darin N. (2009). A population-based study of genotypic and phenotypic variability in children with spinal muscular atrophy. Acta Paediatr. Int. J. Paediatr..

[2] Sugarman E.A., Nagan N., Zhu H., Akmaev V.R., Zhou Z., Rohlfs E.M., Flynn K., Hendrickson B.C., Scholl T., Sirko-Osadsa D.A. (2012). Pan-ethnic carrier screening and prenatal diagnosis for spinal muscular atrophy: Clinical laboratory analysis of >72,400 specimens. Eur. J. Hum. Genet..

[3] Verhaart I.E.C., Robertson A., Wilson I.J., Aartsma-Rus A., Cameron S., Jones C.C., Cook S.F., Lochmüller H. (2017). Prevalence, incidence and carrier frequency of 5q-linked spinal muscular atrophy-A literature review. Orphanet J. Rare Dis..

[4] Jędrzejowska M. (2020). Advances in Newborn Screening and Presymptomatic Diagnosis of Spinal Muscular Atrophy. Degener. Neurol. Neuromuscul. Dis..

[5] Lunn M.R., Wang C.H. (2008). Spinal muscular atrophy. Lancet.

[6] D’Amico A., Mercuri E., Tiziano F.D., Bertini E. (2011). Spinal muscular atrophy. Orphanet J. Rare Dis..

[7] Finkel R.S., Mercuri E., Meyer O.H., Simonds A.K., Schroth M.K., Graham R.J., Kirschner J., Iannaccone S.T., Crawford T.O., Woods S. (2018). Diagnosis and management of spinal muscular atrophy: Part 2: Pulmonary and acute care; medications, supplements and immunizations; other organ systems; and ethics. Neuromuscul. Disord..

[8] Wirth B. (2000). An update of the mutation spectrum of the survival motor neuron gene (SMN1) in autosomal recessive spinal muscular atrophy (SMA). Hum. Mutat..

[9] Prior T.W. (2010). Spinal muscular atrophy: A time for screening. Curr. Opin. Pediatr..

[10] Maretina M.A., Zheleznyakova G.Y., Lanko K.M., Egorova A.A., Baranov V.S., Kiselev A.V. (2018). Molecular Factors Involved in Spinal Muscular Atrophy Pathways as Possible Disease-modifying Candidates. Curr. Genom..

[11] Ramdas S., Servais L. (2020). New treatments in spinal muscular atrophy: An overview of currently available data. Expert Opin. Pharmacother..

[12] Neil E.E., Bisaccia E.K. (2019). Nusinersen: A novel antisense oligonucleotide for the treatment of spinal muscular atrophy. J. Pediatr. Pharmacol. Ther..

[13] Poirier A., Weetall M., Heinig K., Bucheli F., Schoenlein K., Alsenz J., Bassett S., Ullah M., Senn C., Ratni H. (2018). Risdiplam distributes and increases SMN protein in both the central nervous system and peripheral organs. Pharmacol. Res. Perspect..

[14] Stevens D., Claborn M.K., Gildon B.L., Kessler T.L., Walker C. (2020). Onasemnogene Abeparvovec-xioi: Gene Therapy for Spinal Muscular Atrophy. Ann. Pharmacother..

[15] Swoboda K.J., Prior T.W., Scott C.B., McNaught T.P., Wride M.C., Reyna S.P., Bromberg M.B. (2005). Natural history of denervation in SMA: Relation to age, SMN2 copy number, and function. Ann. Neurol..

[16] Govoni A., Gagliardi D., Comi G.P., Corti S. (2018). Time Is Motor Neuron: Therapeutic Window and Its Correlation with Pathogenetic Mechanisms in Spinal Muscular Atrophy. Mol. Neurobiol..

[17] Chien Y.H., Chiang S.C., Weng W.C., Lee N.C., Lin C.J., Hsieh W.S., Lee W.T., Jong Y.J., Ko T.M., Hwu W.L. (2017). Presymptomatic Diagnosis of Spinal Muscular Atrophy Through Newborn Screening. J. Pediatr..

[18] Boemer F., Caberg J.H., Dideberg V., Dardenne D., Bours V., Hiligsmann M., Dangouloff T., Servais L. (2019). Newborn screening for SMA in Southern Belgium. Neuromuscul. Disord..

[19] Lin Y., Lin C.H., Yin X., Zhu L., Yang J., Shen Y., Yang C., Chen X., Hu H., Ma Q. (2019). Newborn Screening for Spinal Muscular Atrophy in China Using DNA Mass Spectrometry. Front. Genet..

[20] Shinohara M., Niba E.T.E., Wijaya Y.O.S., Takayama I., Mitsuishi C., Kumasaka S., Kondo Y., Takatera A., Hokuto I., Morioka I. (2019). A novel system for spinal muscular atrophy screening in newborns: Japanese pilot study. Int. J. Neonatal Screen..

[21] Vill K., Kölbel H., Schwartz O., Blaschek A., Olgemöller B., Harms E., Burggraf S., Röschinger W., Durner J., Gläser D. (2019). One year of newborn screening for SMA–Results of a German pilot project. J. Neuromuscul. Dis..

[22] Czibere L., Burggraf S., Fleige T., Glück B., Keitel L.M., Landt O., Durner J., Röschinger W., Hohenfellner K., Wirth B. (2020). High-throughput genetic newborn screening for spinal muscular atrophy by rapid nucleic acid extraction from dried blood spots and 384-well qPCR. Eur. J. Hum. Genet..

[23] Kay D.M., Stevens C.F., Parker A., Saavedra-Matiz C.A., Sack V., Chung W.K., Chiriboga C.A., Engelstad K., Laureta E., Farooq O. (2020). Implementation of population-based newborn screening reveals low incidence of spinal muscular atrophy. Genet. Med..

[24] Strom C.M., Anderson B., Peng M., Patel U., Braastad C.D., Sun W. (2013). 1000 sample comparison of MLPA and RT-PCR for carrier detection and diagnostic testing for Spinal Muscular Atrophy Type 1. Open J. Genet..

[25] Pan J., Zhang C., Teng Y., Zeng S., Chen S., Liang D., Li Z., Wu L. (2021). Detection of Spinal Muscular Atrophy Using a Duplexed Real-Time PCR Approach With Locked Nucleic Acid-Modified Primers. Ann. Lab. Med..

[26] Pyatt R.E., Prior T.W. (2006). A feasibility study for the newborn screening of spinal muscular atrophy. Genet. Med..

[27] Anhuf D., Eggermann T., Rudnik-Schöneborn S., Zerres K. (2003). Determination of SMN1 and SMN2 copy number using TaqMan^TM^ technology. Hum. Mutat..

[28] Passon N., Pozzo F., Molinis C., Bregant E., Gellera C., Damante G., Lonigro R.I. (2009). A Simple Multiplex Real-Time PCR Methodology for the SMN1 Gene Copy Number Quantification. Genet. Test. Mol. Biomark..

[29] McMillan H.J., Kernohan K.D., Yeh E., Amburgey K., Boyd J., Campbell C., Dowling J.J., Gonorazky H., Marcadier J., Tarnopolsky M.A. (2020). Newborn Screening for Spinal Muscular Atrophy: Ontario Testing and Follow-up Recommendations. Can. J. Neurol. Sci. J. Can. Sci. Neurol..

[30] Wirth B., Herz M., Wetter A., Moskau S., Hahnen E., Rudnik-Schöneborn S., Wienker T., Zerres K. (1999). Quantitative analysis of survival motor neuron copies: Identification of subtle SMN1 mutations in patients with spinal muscular atrophy, genotype- phenotype correlation, and implications for genetic counseling. Am. J. Hum. Genet..

[31] Prior T.W., Snyder P.J., Rink B.D., Pearl D.K., Pyatt R.E., Mihal D.C., Conlan T., Schmalz B., Montgomery L., Ziegler K. (2010). Newborn and carrier screening for spinal muscular atrophy. Am. J. Med. Genet. Part A.

[32] Strunk A., Abbes A., Stuitje A.R., Hettinga C., Sepers E.M., Snetselaar R., Schouten J., Asselman F.L., Cuppen I., Lemmink H. (2019). Validation of a fast, robust, inexpensive, two-tiered neonatal screening test algorithm on dried blood spots for spinal muscular atrophy. Int. J. Neonatal Screen..

[33] Kraszewski J.N., Kay D.M., Stevens C.F., Koval C., Haser B., Ortiz V., Albertorio A., Cohen L.L., Jain R., Andrew S.P. (2018). Pilot study of population-based newborn screening for spinal muscular atrophy in New York state. Genet. Med..

[34] RCoreTeam *R: A Language and Environment for Statistical Computing*; R Foundation for Statistical Computing: Vienna, Austria. https://www.R-project.org/.

[35] Gutierrez-Mateo C., Timonen A., Vaahtera K., Jaakkola M., Hougaard D.M., Bybjerg-Grauholm J., Baekvad-Hansen M., Adamsen D., Filippov G., Dallaire S. (2019). Development of a multiplex real-time PCR assay for the newborn screening of SCID, SMA, and XLA. Int. J. Neonatal Screen..

[36] Cavdarli B., Ozturk F.N., Guntekin Ergun S., Ergun M.A., Dogan O., Percin E.F. (2020). Intelligent Ratio: A New Method for Carrier and Newborn Screening in Spinal Muscular Atrophy. Genet. Test. Mol. Biomark..

[37] Phan H.C., Taylor J.L., Hannon H., Howell R. (2015). Newborn screening for spinal muscular atrophy: Anticipating an imminent need. Semin. Perinatol..

[38] Vidal-Folch N., Gavrilov D., Raymond K., Rinaldo P., Tortorelli S., Matern D., Oglesbee D. (2018). Multiplex droplet digital PCR method applicable to newborn screening, carrier status, and assessment of spinal muscular atrophy. Clin. Chem..

[39] Kellar-Guenther Y., McKasson S., Hale K., Singh S., Sontag M.K., Ojodu J. (2020). Implementing statewide newborn screening for new disorders: U.S. Program experiences. Int. J. Neonatal Screen..

[40] Glascock J., Sampson J., Connolly A.M., Darras B.T., Day J.W., Finkel R., Howell R.R., Klinger K.W., Kuntz N., Prior T. (2020). Revised Recommendations for the Treatment of Infants Diagnosed with Spinal Muscular Atrophy Via Newborn Screening Who Have 4 Copies of SMN2. J. Neuromuscul. Dis..

[41] Müller-Felber W., Vill K., Schwartz O., Gläser D., Nennstiel U., Wirth B., Burggraf S., Röschinger W., Becker M., Durner J. (2020). Infants Diagnosed with Spinal Muscular Atrophy and 4 SMN2 Copies through Newborn Screening-Opportunity or Burden?. J. Neuromuscul. Dis..

[42] Kemper A.R., Lam K., Comeau A.M., Kwon J., Green N.S., Ojodu J., Grosse S., Prosser L.A., Jones E., Tanksley S. (2018). Evidence-Based Review of Newborn Screening for Spinal Muscular Atrophy (SMA): Final Report (v5.2).

[43] Dangouloff T., Servais L. (2019). Clinical evidence supporting early treatment of patients with spinal muscular atrophy: Current perspectives. Ther. Clin. Risk Manag..

[44] Arnold W.D., Kassar D., Kissel J.T. (2015). Spinal muscular atrophy: Diagnosis and management in a new therapeutic era. Muscle Nerve.

[45] Keinath M.C., Prior D.E., Prior T.W. (2021). Spinal muscular atrophy: Mutations, testing, and clinical relevance. Appl. Clin. Genet..

