# Supplementary material for: Newborn Screening for 5q Spinal Muscular Atrophy: Comparisons between Real-Time PCR Methodologies and Cost Estimations for Future Implementation Programs
Source: Int J Neonatal Screen. 2021 Aug 11;7(3):53. doi: 10.3390/ijns7030053 (PMC8396021; doi:10.3390/ijns7030053)
Supplement: Supplementary file 1 [file IJNS-07-00053-s001.zip › IJNS-1247465-supplementary.pdf]

SUPPLEMENTARY MATERIAL

Methods

Table S1. Study cohorts specified by inclusion criteria, total number, sample source, method of DNA extraction, and real time PCR assay performed.

	Inclusion Criteria	N	Sample Source	Method of DNA extraction	Real time PCR assay performed
**Retrospective Cohort**	Individuals with *SMN1/SMN2* genotype previously obtained by MLPA and recorded at our center	54	Peripheral blood collected in 4 mL tube containing EDTA	Autopure LS (Qiagen/Gentra, Hilden, Germany)	P/A (in-house)
**Prospective Cohort**	Individuals with clinical hypothesis of SMA, SMA relatives and possible carriers, and non-related and non-SMA individuals	75	Peripheral blood collected in 4 mL tube containing EDTA	Biorobot QIAsymphony (Qiagen, Hilden, Germany)	P/A (in-house)
**Mixed Cohort**	Individuals either from the retrospective or prospective cohort randomly assigned	26	Blood collected on the S&S 903 filter paper	NaOH protocol, as described in Strunk et al. 2019	P/A (in-house)<br>Melting curve (commercial)

MLPA – Multiplex ligation-dependent probe amplification; SMA – Spinal Muscular Atrophy; P/A – Presence/Absence assay;

Strunk, A.; Abbes, A.; Stuitje, A.R.; Hettinga, C.; Sepers, E.M.; Snetselaar, R.; Schouten, J.; Asselman, F.L.; Cuppen, I.; Lemmink, H.; et al. Validation of a fast, robust, inexpensive, two-tiered neonatal screening test algorithm on dried blood spots for spinal muscular atrophy. *Int. J. Neonatal Screen.* **2019**, *5*, 1–9, doi:10.3390/ijns5020021.

Results

Clinical follow up and *SMN2* copy number

Among 20 patients included in the study with clinically suspected SMA (prospective cohort; see methods in the main tect), MLPA confirmed the molecular diagnosis in 18 participants: 5 of them presented with *SMN1* exon 7 deletion, but not exon 8. Seventy-two percent of the SMA molecularly confirmed patients presented with 3 *SMN2* copies and SMA type II or III phenotype (Figure S1).

Clinical evaluation was reviewed for the 2 patients with negative MLPA results. Electroneuromyography was suggestive of distal spinal atrophy or peripheral polyneuropathy in one of them, excluding the clinical hypothesis of 5q SMA. This sample was included in the Next Generation Sequencing (NGS) panel of motor and sensitive diseases of the peripheral nervous system, but the cause of the clinical findings remains unknown. The other patients with negative MLPA results, presented with some features of SMA along with facial dimorphisms. Further investigation revealed a heterozygous variant in the *DYNC1H1* (c.3581A>G p.(Gln1194Arg), hg38), also excluding the 5q SMA hypothesis.

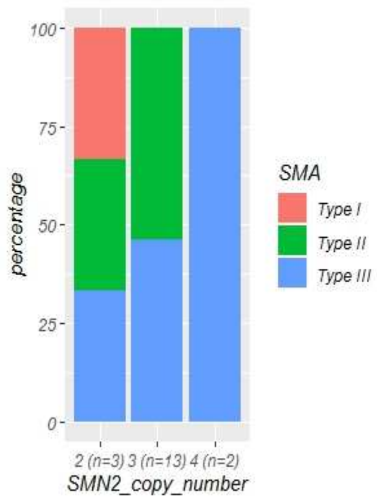

Figure S1. Distribution of SMA types according to the number of *SMN2* copies.
